# Electrochemical Characterization of Polymeric Coatings for Corrosion Protection: A Review of Advances and Perspectives

**DOI:** 10.3390/polym14122306

**Published:** 2022-06-07

**Authors:** Andressa Trentin, Amirhossein Pakseresht, Alicia Duran, Yolanda Castro, Dušan Galusek

**Affiliations:** 1 FunGlass, A. Dubček University of Trenčín, Študentská 2, 911 50 Trenčín, Slovakia; amir.pakseresht@tnuni.sk (A.P.); dusan.galusek@tnuni.sk (D.G.); 2 Instituto de Cerámica y Vidrio (CSIC), Campus de Cantoblanco, 28049 Madrid, Spain; aduran@icv.csic.es (A.D.); castro@icv.csic.es (Y.C.); 3 Joint Glass Centre of the IIC SAS, TnUAD and FChFT STU, 911 50 Trenčín, Slovakia

**Keywords:** polymeric coatings, anti-corrosion, corrosion inhibitor, electrochemistry

## Abstract

The development of anti-corrosion polymeric coatings has grown exponentially in the fields of material science, chemistry, engineering, and nanotechnology during the last century and has prompted the evolution of efficient characterization techniques. Nowadays, polymeric coatings represent a well-established protection system that provides a barrier between a metallic substrate and the environment. However, the increase in complexity and functionality of these coatings requires high-precision techniques capable of predicting failures and providing smart protection. This review summarizes the state of the art for the main electrochemical techniques, emphasizing devices that track the anti-corrosion properties of polymeric coatings from the macroscale to the nanoscale. An overview of the advances in accelerated corrosion testing and the electrochemical characterization of coatings is explored, including insights into their advantages and limitations. In addition, the challenges and potential applications of the theoretical approaches are summarized based on current knowledge. Finally, this work provides the reader with the trends and challenges of designing future technologies and models capable of tracking corrosion and predicting failures.

## 1. Introduction

The history of corrosion research dates back to 1910, to studies of non-ferrous metals used in marine conditions [1]. The lack of knowledge about the phenomenon of corrosion during World War I in 1914 brought difficulties to the Royal Navy, caused by the failure of brass condenser tubes. Many important studies were conducted by researchers in the USA and in European institutes in the following decades, providing new frontiers for the understanding of corrosion mechanisms [1]. However, monitoring corrosive processes through methodical scientific inspection became mandatory with the sharp increase in the production of automobiles and aircrafts. This boosted the development of new techniques, other than visual, gravimetric, and accelerated corrosion inspection, that could accurately detect the appearance of localized forms of corrosion (e.g., pitting) [2]. Discoveries of the fundamentals of metallic corrosion during this period were essential for the establishment of the main analysis methods, which were later applied to polymeric coatings. In addition, the development of tests simulating conditions close to those encountered during usage made it possible to transfer knowledge about metallic corrosion to the evaluation of coating performance [3].

A few years earlier, the work of Nernst, in 1894, represented a milestone for studies on the dielectric properties and resistance of galvanic cells by compiling the knowledge obtained by Wheatstone regarding aqueous media. Additional developments obtained in the following century, such as oxide studies, diffusion measurements, double electrical layers, and fitting by electrical circuits, were essential for the consolidation of the role of electrochemical measurements in the study of thin films and coatings [4]. The use of potential-time or open-circuit potential curves (OCP) to study the protection afforded by coated metals dates back more than 80 years [5]. It was introduced by Burns and Haring in 1936 by employing the knowledge obtained by Evans on metal oxides [6]. The authors evaluated painted iron using a potentiometer and reached the conclusion that protection by iron oxide is physical and that by red lead (pigment and primer) is chemical [6]. Originally, the researchers tried to reduce the prolonged exposure time in field tests and they observed that the use of potential-time assays was a promising alternative for failure prediction [7]. Observations of potential differences of at least 0.5 V between the painted and non-coated surfaces suggested that the method was an efficient tool for quick evaluations of coatings [6].

In 1938, the pioneering work of Wagner and Traud linked the electrochemical tests with the studies on corrosion, drawing special attention to polarization curve measurements. However, this analysis was not yet applied to coatings [8,9]. Nearly simultaneously, the need to locate the corroded areas in pipelines indicated the urgency for new measurement techniques and for barrier coatings, accelerating the fusion of two sciences: electrochemistry and polymeric coatings. For instance, with the use of the Pearson Holliday detector in 1941, it was possible to carry out field measurements of current flows between tubes and the ground, thus identifying the location of coatings defects with precision [10]. In a similar configuration, a McCollum ground current meter was employed in pipe-to-soil potential tests connecting a battery, voltmeter, and millimeter in 1952 [11]. The apparatus allowed the identification and location of corrosion sites in coatings through the potential drop along the length of the tube.

A milestone in coatings analysis was achieved in 1948 by the extensive work of Bacon et al. [12]. The authors investigated over 300 systems with a potentiometer and found a reliable correlation between the coatings’ corrosion protection and resistance values. Good protection was found for films with resistances higher than 100 MΩ cm^−2^, while poor protection was obtained for resistances below 1 MΩ cm^−2^ [12,13,14]. From then on, polarization and potentiometric studies became widely used for the practical evaluation of corrosion protection [5]. In 1960, the third international exhibition of electronic instruments and automation drew the research community’s attention to corrosion studies using portable devices. Portable voltmeters and potentiometers developed by Cambridge Instruments and Cathodic Control Ltd. provided the needed features to resist rough handling in the field [15]. 

It was only in 1972, however, that Takenouti et al. shed light on the application of electrochemical impedance spectroscopy (EIS) tests for studies of anti-corrosion coatings [4,16]. This work was a milestone in making EIS the most suitable technique to monitor the permeation and collapse of immersed samples in long-term tests. Through a critical examination of the electrochemical methods used to assess the rate of metallic corrosion, the authors established a direct relationship between charge transfer resistance, the rate of corrosion, and corrosion resistance [16]. As a non-destructive ac technique, EIS provides quantitative data on the performance of a coating and can indicate water uptake long before any visible change [17,18]. Therefore, the evolution of resistance values associated with electron transfer reactions (corrosion) and capacitance, representing the swelling of coatings, started being used to estimate a coating’s failures [17,19,20]. Some years later, the work of Rowlands and Chuter, in 1983, was one of the first to report the application of EIS measurements to track paint system breakdown. However, some issues such as frequency range, solution resistance, electrode distance, use of an auxiliary electrode, and risk of electrode polarization after open-circuit potential measurements remained practical problems yet to be addressed [19].

**Figure 1:**
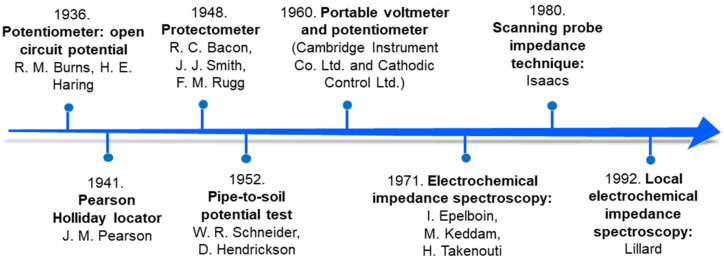
Timeline of milestones in advances of polymeric coating characterization [6,10,11,12,15,16,18,21].

New devices were developed following the evolution of techniques based on electrochemical knowledge. Surface inhomogeneities of coatings were first analyzed by Isaacs and Kendig in 1980 using a scanning probe impedance technique. The promising results achieved in the first tests marked the beginning of a new era in coating characterization: local electrochemical measurements [21]. The scanning probe impedance allowed the detection of spatial impedance variations, meaning the rates of electrochemical reactions in the surface heterogeneities of the coatings could be measured [21]. This advance boosted the development of local electrochemical impedance spectroscopy (LEIS) in the following years (1992) by Lillard et al. [18]. In this approach, the authors were able to map the alternating current (AC) density of the working electrode surface in three dimensions, allowing heterogeneities to be tracked with precision [18]. Some important milestones in the development of polymeric coating characterization are summarized in Figure 1.

**Figure 2:**
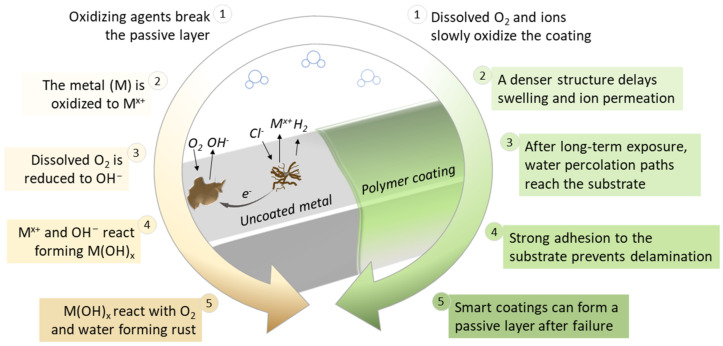
A representative cycle of metallic corrosion (left) and protection of typical polymeric coatings (right) immersed in aerated neutral saline solution [13,27,36].

The knowledge obtained in one century of studies in corrosion science and coating technologies can be summarized in five steps. They are depicted in Figure 2 through the cycle of metallic corrosion (left) and the protection mechanism provided by polymeric coatings (right) in passive (steps 1–4) and active (step 5) systems. Figure 2 shows that an uncoated metal corrodes when in contact with a humid atmosphere or electrolyte aqueous solution. This process involves metal oxidation in the anodic region forming ionic species and electrons which are consumed in the oxygen reduction reaction in the cathodic region [22]. As a result of hydrolysis of cations and generation of protons, the electrolyte at the bottom of the pit becomes positively charged, attracting chlorine ions (Cl^−^) which react with the metal ions formed in the previous step, resulting in insoluble products and hydrochloric acid, causing further pH decrease at the anode from 6 to 2–3 [22,23,24]. Simultaneously, the electrons are transferred to the cathodic sites and react with the dissolved oxygen to form hydroxyl groups, increasing the local pH [22,25,26]. Finally, metallic ions and OH- combine and produce insoluble hydroxides or react with oxygen and water to form rust. Typically, if the anode and cathode sites move continuously across the surface, widespread corrosion occurs [22].

The application of coatings, in turn, prevents corrosion by providing a passive layer with only gradual permeation by water and ions (Figure 2, right) [27]. The denser the structure, the slower the oxidative attack of dissolved ions, ensuring years of corrosion protection even for coatings with thicknesses of a few micrometers [27,28,29,30,31,32]. However, even very good barriers undergo water uptake to some extent after contact with water/electrolytes. At this stage, the adhesion of the coating to the substrate plays a crucial role in avoiding delamination and consequent corrosion expansion [27]. After water/electrolytes permeate the structural flaws of the coatings and/or reaches the metal interface, smart coatings that contain corrosion inhibitors or self-healing agents can affect the interface in terms of charge transfer and the formation of a passive film or by restoring the barrier properties, thus extending the protection and the coatings’ service life [33,34,35,36].

The characterization of passive and active coatings includes in-depth knowledge of structure, morphology, and composition, along with the barrier properties assessed by conventional and localized electrochemical testing. To this end, new technologies and devices are developed every year to test complete coating systems, representing a key factor in ensuring the quality of coatings. When considering the electrochemical assessment of coatings, the methods can be broadly categorized as conventional and advanced (localized) electrochemical measurements. Another important approach to investigate the barrier and active properties of polymeric coatings consists of accelerated corrosion tests.

**Figure 3:**
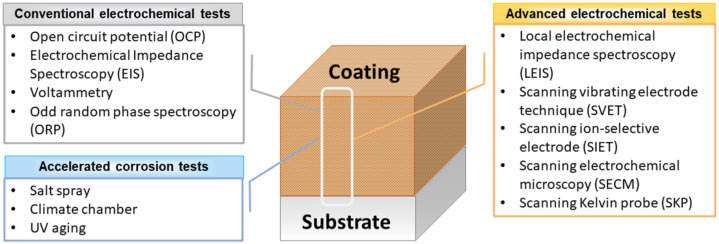
Overview of the main electrochemical techniques and accelerated corrosion tests currently used to characterize polymeric anti-corrosion coatings.

Self-healing/smart coatings have shown exponential development in the last few years. Since the work of White et al. in 2001 [37], several strategies have been exploited aiming to meet the demanding needs of the coatings market, providing consumers with multifunctional materials that can protect metallic surfaces with the same performance as the conventional ones [36,38]. More specifically, a self-healing coating must be able to recover its barrier properties after damage with or without external stimuli, extending the lifetime and safety of coated surfaces [36,38]. Some approaches stand out for active anti-corrosion protection, regardless of the type of polymeric matrix, such as (i) corrosion inhibitors (organic and inorganic compounds) [39], (ii) chelation systems based on coordination bonds [40], (iii) polymers with shape-memory properties [41], (iv) reversible processes based on Diels–Alder (DA) and retro-Diels–Alder reactions [42], (v) dynamic imine bond formation and enzymatic polymerization [43], (vi) microcapsules containing monomer and catalyst [44], and (vii) interconnected vascular networks [45]. Nonetheless, all these advances have brought new challenges in terms of the devices capable of detecting self-healing, resulting in electrochemical techniques capable of identifying local fault repair (Figure 3, right).

Figure 3 presents a scheme of the main electrochemical techniques and accelerated corrosion tests employed in the characterization of polymeric or hybrid coatings, considering the extent of the measurement in terms of surface, bulk, and substrate.

Nowadays, assessing the real efficiency of anti-corrosion polymeric coatings has become crucial to develop polymers, paints, varnishes, binders, crosslinkers, pigments, and solvents that maximize the service life and safety of pipelines, automobiles, aircraft, trains, ships, just to name a few applications. Considering the presented historical advances, the following sections describe successful examples, advantages, and limitations of the main electrochemical techniques currently used to analyze anti-corrosion polymeric coatings. The wide applicability of electrochemical tests in this field is based on the fast measurements obtained with relatively simple instrumentation. In addition, accurate quantitative or semi-quantitative results are obtained when evaluating film protection [46]. The abilities to analyze coatings in situ and to track failures make conventional and localized electrochemical techniques an excellent choice among industrialists, engineers, and researchers. 

Finally, although their use and applicability are well-resolved issues in laboratories and industries, some challenges arise when it comes to long-term field evaluation, study of corrosion inhibitors in a reproducible way, prediction of failures before emergence and the replacement of toxic compounds for bio-based alternatives. These challenges were also summarized by a review on electrochemical measurements used for corrosion assessment, where the authors considered the complexity of real systems, pointing to easier, reliable, and non-destructive techniques as the most suitable for in situ analysis [47]. From this perspective, this work provides a concise yet comprehensive compilation of the milestones in the analysis of polymeric coatings, focusing on successful examples of the electrochemical methods used to evaluate smart/self-healing polymeric coatings. The theoretical approaches covered in the last section highlight trends and challenges for the future design of technologies and models capable of tracking corrosion and predicting failures.

## 2. Characterization of Polymeric Coatings

Selecting the proper method for the evaluation of coated metals is not trivial and represents a key step in the development of high-performance materials. Considerable efforts have been made since the early 1900s to design techniques that simulate real-world conditions [48]. Without the ambition of being extensive, this review covers three main branches in the analysis of coatings, namely (i) accelerated corrosion tests, (ii) conventional electrochemical analysis, and (iii) advanced electrochemical analysis. While the first and second sections explore the use of the salt-spray test, open-circuit potential (OCP), and electrochemical impedance spectroscopy (EIS), the third section deals with localized electrochemical impedance spectroscopy (LEIS), scanning vibrating electrode technique (SVET), scanning ion-selective electrode technique (SIET), scanning Kelvin probe (SKP), and scanning electrochemical microscopy (SECM). Other relevant electrochemical methods mainly used in the characterization of conductive polymeric coatings, such as polyaniline, polypyrrole, or polythiophene [49,50], include cyclic voltammetry, chronoamperometry, potentiodynamic, potentiostatic, and galvanostatic techniques [51,52,53].

### 2.1. Accelerated Corrosion Tests

Accelerated corrosion tests are employed in many studies by engineers and materials scientists to speed up the degradation process and to understand the reactions that occur in metals coated with polymeric coatings [48,54]. The main practices include the salt-spray test (continuous salt fog) and cyclic corrosion test, also known as Prohesion test (wet-dry intervals) [55]. Although the lack of correlation to field studies has been the subject of debate, specifications such as those of the American Society for Testing and Materials (ASTM) have been adapted accordingly and are widely employed by laboratories and industries [48].

#### Salt-Spray Tests

Salt spray, also known as the salt fog test, consists of uninterrupted exposure of intact or damaged coated substrates to a salty fog solution (5% sodium chloride) in a chamber. Parameters such as temperature, pH, air pressure, duration of the test and UV radiation are pre-determined by the operator [55]. The salt-spray test has undergone several modifications so that laboratory assays are comparable with field tests, usually carried out over 5 years [54]. One of the main advantages includes its relatively inexpensive and easy procedure that provides quick comparative results. One must take into account, however, that this qualitative analysis does not represent real-world conditions and besides destructive, it provides results for saline environments only.

It is known that ultraviolet (UV) radiation, humidity, temperature, pH, and the presence of oxygen and salt are the main driving forces that contribute to the onset of corrosion and the failure of films [56,57]. Factors that affect the reproducibility of results, such as the effects of pH, fog sedimentation rate, air pressure, temperature, sample position, solution concentration, etc., were established after extensive research and are currently used by the suppliers of these devices [54]. The evolution of ASTM specifications to the current B117 has provided the necessary practices to develop salt-spray chambers and accessories that allow for the control of accelerated corrosion tests [57]. For this purpose, some conditions must be fulfilled, namely (i) the same corrosion mechanism as in real-world conditions, (ii) the relationship between measured and varied parameters, and (iii) the same probability of the results distribution [58].

**Figure 4:**
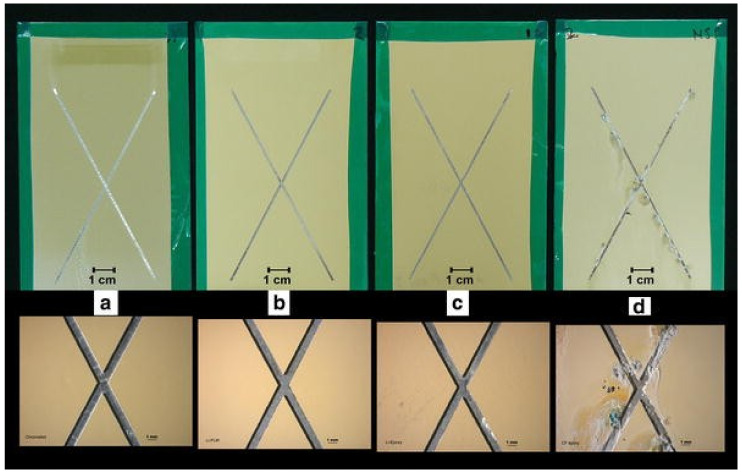
Optical images of AA2024-T3 aluminum alloy coated with industrial coating concepts after 3000 h salt spray exposure (top, entire panel; bottom, increased magnification): (**a**) chromate primer, (**b**) polyurethane-based primer with lithium salt inhibitor technology, (**c**) epoxy-amine-based primer with lithium salt inhibitor technology, and (**d**) a previous generation chromate-free inhibitor technology. Reproduced with permission of Springer Nature [59].

Salt-spray tests are particularly useful for investigating the mechanism of action for corrosion inhibitors loaded into intact or scribed coatings. Interesting results were found by comparing the activity of inhibitors based on chromates, lithium, and other chromate-free substances [59]. Figure 4 shows optical images of AA2024 aluminum alloy coated with a chromate-based primer, commercial polyurethane, and epoxy-amine primers modified with lithium and chromium-free corrosion inhibitors after 3000 h exposure to salt spray. The systems containing chromium and lithium had similar performance, with almost no corrosion products after the test, while the other inhibitors showed severe corrosion in the scratch region (Figure 4d) [59]. The comparison between the different polymers showed that the polyurethane-based film promoted a slight improvement in corrosion protection [59].

Combined salt-spray and EIS analysis revealed that a waterborne epoxy composite coating modified with polypyrrole and exfoliated graphene tends to develop a metal oxide layer on the interface coating/steel [53,60]. This is due to a dual effect: (i) the good dispersion of graphene promoted by the polypyrrole that creates a physical shielding effect like a “labyrinth” for intact coatings and (ii) the reduction of electrons released in the corrosion process by the conducting polymer extending the protection of damaged coatings [53,60]. Similar results were observed for sodium–zinc–molybdate nanoparticles incorporated into graphene–polyaniline epoxy coatings for the corrosion protection of mild steel [61]. The salt-spray test showed that the nanocontainers formed a passivating layer with good adherence to the substrate since no presence of rust was found on the panels even after 500 h of testing (ASTM B-117) [61]. The inhibitive effect of polyaniline/CeO_2_/epoxy nanocomposites on carbon steel was confirmed through potentiodynamic polarization curves and salt-spray tests [53,62]. A hydrothermal route followed by in situ polymerization was used to synthesize the polyaniline/CeO_2_ NPs. The enhanced corrosion protection found in this system was attributed to a synergetic effect of the diffusion barrier and redox properties of polyaniline [62]. 

Duong et al. performed a salt-spray study of hydrotalcite-containing epoxy coatings modified with 2-benzothiazolythio-succinic acid (BTSA) and benzoate (BZ) as the inhibitors. The results reveal the potential of corrosion inhibition when 1.5% BZ is added to the material [63]. The evaluation of the coatings after 96 h of salt-spray exposure shows a lower degree of delamination and formation of corrosion products when the inhibitor is present compared to the unmodified epoxy coating. The authors attributed this effect to the low solubility of the benzoate in water and the more intense leaching in the scratched area, limiting the spread of corrosion [63]. Other examples of accelerated corrosion tests applied to self-healing coatings include immersion in 3.5 wt.% NaCl solution (NACE/ASTM TMO169/G31) for 75 days [64], the Q-panel condensation (QCT) test performed at 41° C, continuous condensation without drying (ASTM D 4585-99) for 1000 h [65], or the Prohesion cyclic test for 4896 h (ASTM G85-09), which is comprised of successive drying cycles and salt concentration variation [66].

### 2.2. Conventional Electrochemical Analyses

Electrochemical tests are the state of the art in the evaluation of anti-corrosion coatings. The conventional methods are considered efficient tools to monitor the degradation of an average of the electrochemical processes taking place on the surface of the working electrode. Some of the main practices in coatings evaluation include open-circuit potential (OCP) and electrochemical impedance spectroscopy (EIS). Other common tests can be mentioned such as linear polarization resistance (LPR), electrochemical noise (EN), electrochemical frequency modulation (EFM), potentiostatic step polarization, and galvanostatic step polarization [47]. Nonetheless, the next sections describe the principles, examples, advantages, and limitations of OCP and EIS.

#### 2.2.1. Open-Circuit Potential (OCP)

Open-circuit potential is the potential of a working electrode when no current is applied to the cell [46]. Tracking the OCP is a useful practice for monitoring the degradation of barrier coatings, since the passage of electrons through the coating causes the OPC value to drop to the metal substrate [46]. It is known that more negative potentials in time-potential measurements may indicate the removal of oxide layers from the surface and initiation of corrosion, while more positive potentials indicate the beginning of a protective film formation. However, the corrosion potential itself can be an erroneous indicator of corrosion as nobler potentials do not necessarily suggest slower corrosion rates [5]. This was explained by the pioneering work of Evans and later by Pourbaix using potential-pH diagrams, where a positive shift of pH within an active corrosion zone increases the corrosion rate, not the opposite [5,8]. In conclusion, electrochemical measurements correlated to the results of accelerated corrosion tests are found to be more reliable and reproducible.

**Figure 5:**
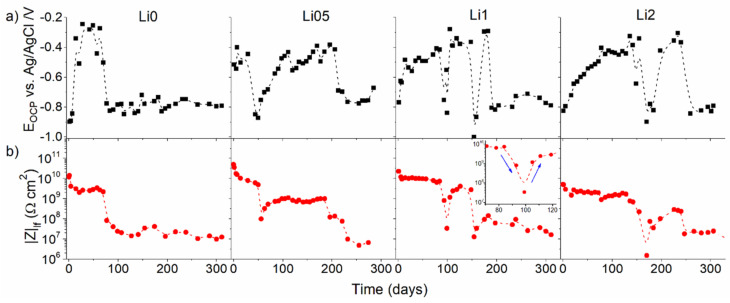
(**a**) Time evolution of EOC and (**b**) |Z|_lf_ for coatings modified with different amounts of lithium during 310 days of immersion in 3.5% NaCl solution. The inset shows the first impedance modulus recovery (99 days) for the Li1 sample in more detail. Reproduced with permission of ACS [33].

In potentiometry, the potential of an electrode is measured in relation to another standardized electrode. This is known as the open-circuit potential or corrosion potential (OCP or E_OC_) [46]. OCP measurements are typically employed before EIS measurements to verify the system’s stability. The main advantages of the technique rely on its simple setup that provides the user with a semi-quantitative evaluation of coatings which can be associated with electrochemical impedance spectroscopy results. Although their values do not provide a direct estimate of corrosion protection, some studies report a direct correlation with low-frequency modulus values of electrochemical impedance measurements (|Z|_lf_) [33] or pore resistance (R_po_) values [67]. The repassivation cycles of substrates by conducting coatings containing corrosion inhibitors can be easily identified by OCP [68]. Figure 5 shows an example comparison of the evolution of OCP and |Z|_lf_ values over the immersion time (310 days) of poly(methyl methacrylate) (PMMA)-silica coatings modified with lithium salts. This study shows the self-healing activity for all compositions containing the inhibitor [33].

#### 2.2.2. Electrochemical Impedance Spectroscopy (EIS)

The possibility of estimating coating barrier characteristics, water uptake, the presence of defects, interface reactivity, and coating adhesion, along with the assessment of parameters such as the coating delamination index, coating damage index, low-frequency impedance, breakpoint frequency, and high-frequency phase angle has made EIS one of the most powerful tools for evaluating the performance of coatings [7,17,69,70]. Overall, EIS is an extremely useful technique to measure and monitor the rate of deterioration/swelling of polymeric coatings exposed to electrolytes and to study electrochemical cathodic and anodic reactions when corrosion occurs. Some of the main advantages of EIS in the study of polymeric coatings are its characteristics of being non-destructive, assessing the degradation or regeneration of films, and most importantly, providing quantitative values for the electrochemical processes taking place in the system [71]. Nonetheless, the results correspond to an average of the surface, and modeling and interpreting diffusing or corroding systems can be complex.

**Figure 6:**
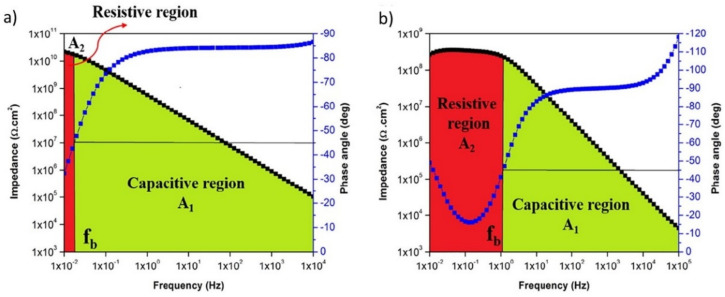
Representative Bode plots of an epoxy–polydimethylsiloxane (ES0) coating after (**a**) 1 day and (**b**) 30 days of immersion, along with the determination of the breakpoint frequency and the corresponding capacitive (A1) and resistive (A2) regions. Reproduced with permission of Elsevier [72].

To obtain quantitative information on the electrochemical processes taking place at the coating–oxide–metal interface it is common to combine resistors and capacitors to represent the behavior of the material in the form of an electrical equivalent circuit. The resistance can represent the electron transfer reactions that occur during corrosion [17]. However, the concept of resistance introduced by Ohm’s law (*R = V/I*, where *R* is the resistance in ohms, *V* is the voltage in volts, and *I* is the current in amperes) is limited only to the resistor and it does not apply to complex, real-world systems. To contour this problem, the impedance formalism (*Z*) is applied. *Z* is the tendency of a circuit to resist the flow of an alternating electric current [71] and is mathematically represented by the expression *Z = V_ac_/I_ac_*. Typically, a sinusoidal voltage disturbance is applied to the system in the range of ±10 mV, resulting in sinusoidal current waves with a phase shift between them, known as φ. The impedance is mostly represented either in the Nyquist format (Z_imaginary_ vs. Z_real_) as Bode plots (ǀZǀ vs. frequency (*f*)) and/or as a phase angle plot ((Φ) vs. *f*) (e.g., Figure 6) [7,17,69].

As the coating deteriorates with the uptake of water and ions, the film presents more resistive features. These features are identified by more stable values of the open-circuit potential and by a decrease in the impedance modulus and phase angle values at low frequencies (*f* < 1 Hz) [17]. Figure 6 shows the variations of the resistive and capacitive regions in Bode plots for an epoxy–polydimethylsiloxane hybrid coating (labeled as ES0, 73 µm thick) applied on mild steel panels after 1 day and 30 days of immersion in 3 wt.% NaCl solution [72]. The stability of the coating can be assessed by the shape of the impedance modulus and the phase angle graphs as a function of frequency. The expansion of the resistive region at low frequencies indicates electrolyte diffusion and a consequent loss of adhesion at the interface with the substrate due to the polymer swelling and corrosion reactions taking place [72].

**Figure 7:**
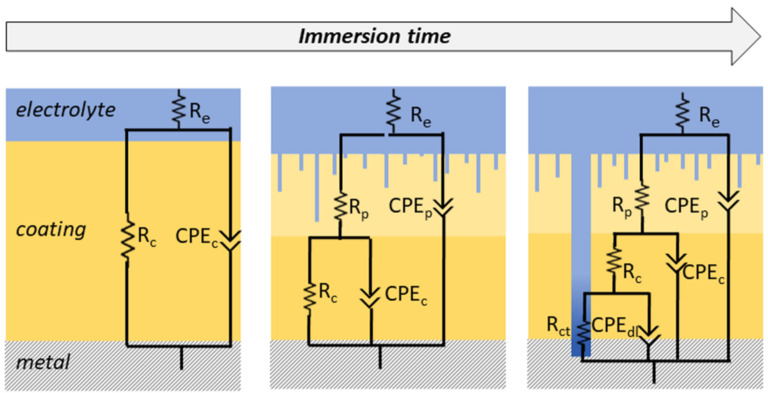
Representative schemes of electrical equivalent circuits (EECs) most employed to fit EIS data of polymeric coatings over immersion time.

When in contact with a solution under an alternating voltage, a good coating can be described as a capacitor (a non-conductive physical barrier between metal and electrolyte) [15,46]. High-performance coatings usually exhibit highly capacitive behavior in the early stages of immersion, acting almost as an ideal capacitor. This behavior depends on the thickness and dielectric constant values of the coating [17]. Nevertheless, electrolyte absorption leads to an uneven distribution of electrical properties across the thickness, resulting in deviations from simple capacitive behavior. Taking into account the non-ideality of the system, a constant phase element (CPE) is normally used during modeling by electrical equivalent circuits (EEC) to replace the capacitor, providing a good correlation with experimental data [28]. The changes in the coating structure caused by the swelling of the polymeric chains are reflected in the physical properties of the material and, consequently, in the EECs that best describe the behavior of the immersed coating. Depending on the reactions taking place in the film, the circuits used to describe the systems can vary. Among many proposed models, some have been widely used to represent an intact coating, a system with pores, and a film with corrosion pits. Figure 7 depicts the main models used to fit the EIS data obtained at different stages of immersion.

**Figure 8:**
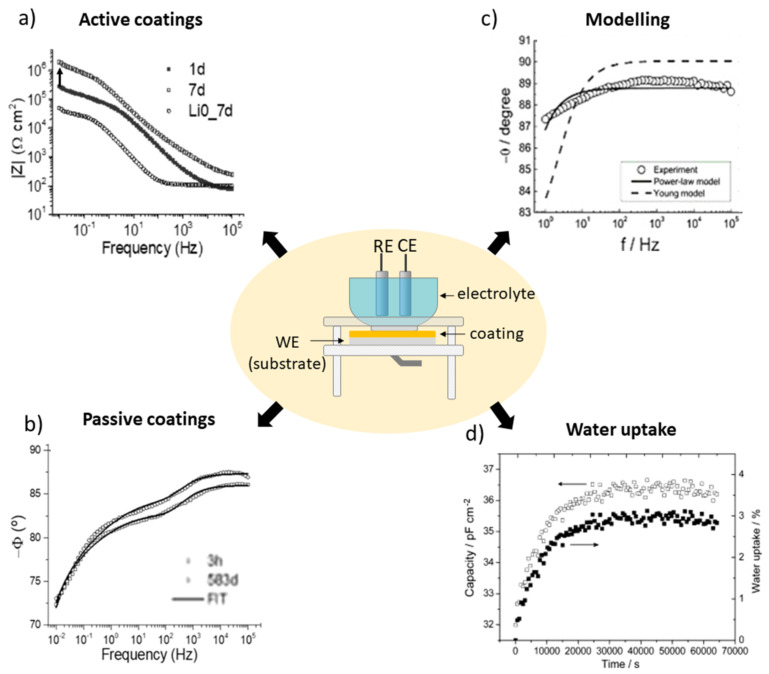
Illustrative scheme of the main applications of electrochemical impedance spectroscopy (EIS) analysis in the field of polymeric anti-corrosion coatings, including (**a**) active coatings, (**b**) passive coatings, (**c**) modelling and (**d**) water uptake calculations. Reproduced with permission of ACS and Elsevier. Adapted from references [28,33,74,76].

In the case of short-time immersion in an electrolyte, the properties of a barrier coating remain intact. In this period, the electrolyte resistance (*R_S_*), the capacitance or CPE (*CPE_C_*), and the resistance of the coating (*R_C_*) are suitable to represent the material (Figure 7, left) [71]. However, this condition remains valid for only a few minutes or seconds of immersion, depending on factors such as thickness, composition, and the dielectric constant of the coating [73,74]. Then, the electrolyte percolates, forming pores or channels and nano- or macro-structural defects. It is then necessary to add a second time-constant to the EEC that represents an outer layer with the presence of pores (*R_P_* and *CPE_P_*) and a preserved inner layer of high impedance (*R_C_* and *CPE_C_*) [28,73]. Finally, the coating failure due to pitting determines the lifespan of the film. At this stage, a third time-constant can be used to describe the behavior of the damaged material. The oxidation–reduction reactions taking place at the coating/metal interface can be described by charge transfer resistance (*R_CT_*) in parallel with the capacitance of the electrical double layer (*C_DL_*) (Figure 8, right) [33,69,75].

It is worthy to mention, however, that EIS tests by themselves do not represent a method for the unique and final analysis of protective coatings. To fully understand the system, the electrochemical measurements must be combined with structural, mechanical, and thermal investigations. For example, Fréchette et al. evaluated six different systems by combining accelerated corrosion tests in salt-spray chambers and Kesternich cabins with EIS tests, FTIR measurements, and adhesion tests [7]. They concluded that the joint analysis favored the understanding of the real-world conditions responsible for the degradation processes, providing valuable information about the best protective material for steel, namely epoxy and polyurethane coatings [7].

Relevant works in the field using the EIS technique have yielded significant discoveries. Among the main applications, studies of passive and active coatings also cover modeling to obtain physical parameters and water uptake calculations. Some interesting examples are shown in Figure 8. EIS proved to be an effective technique to confirm the self-healing activity of PMMA–silica coatings (6.3 µm) containing lithium carbonate as a corrosion inhibitor on AA7075, Figure 8a [33]. The impedance modulus plot shows regeneration of one order of magnitude for artificially scratched coatings containing lithium and subjected to the salt-spray test for 7 days [33]. Figure 8b shows an application of EIS when used to monitor the slow degradation of high-performance passive coatings immersed for 583 days in 3.5 wt.% NaCl solution [28]. The slow rate of electrolyte permeation is almost imperceptible in the impedance modulus graph. However, it can be observed in the phase graph at high frequencies [28].

Other relevant uses of EIS testing include modeling to extract physical parameters. In Figure 8c, the phase plot obtained for polyaminoamide–epoxy coatings (21 µm) on AA2024 compares the experimental data obtained for a dry coating to the regression results using the power law (solid line) and the Young model (dashed line) [74]. The input values for the best fit obtained by the power law include *α*, *ε_c_*, *ρ_c_*, and *ρ_δ_*, where *α* is the CPE exponent, *ε_c_* is the coating permittivity, *ρ_c_* is the coating resistivity, and *ρ_δ_* represents the coating resistivity at the interface with the solution, where the water uptake takes place; *g* is a numerical coefficient with a value very close to 1 when α is close to 1. EIS data were regressed applying Equation (1) [74]:

(1) $$Z\left(\omega \right)=g\frac{\delta \rho_{\delta }^{\left(1-\alpha \right)}}{{\left(\rho_{c}^{-1}+j\omega \varepsilon_{c}\varepsilon_{0}\right)}^{\alpha }}$$

Using this approach, the authors assessed relevant physical properties of the system, such as the resistivity variation with the thickness, while also providing a comparative study on the methods to obtain the best fit. Figure 8d shows the calculation of the capacitance and water uptake (*φ*) of epoxy–amine coatings on n-doped silicon substrates using Equations (2) and (3), respectively [76]. *C_p_* represents the capacitance of the film at time *t*, *C_p0_* is the capacitance of the film extrapolated to *t* = 0, *Z_i_* is the imaginary part of the EIS data obtained at a frequency *f*, and *ε_ω_* is the dielectric constant of water (*ε_ω_* = 78.3) [76]. While almost no visible change was observed in the Bode diagrams, this comparative study revealed that the saturation of the film (~2.5 to 3%), represented by the capacitance and water uptake values, occurred after 15,000 s (~4 h). These values, obtained using the Brasher/Kingsbury equation, correspond to a plateau in the capacitance vs. time curve (Figure 8d) [76].

(2) $$C_{p}=\frac{1}{2\pi fZ_{i}}$$

(3) $$\varphi =\frac{log\left(C_{p}/C_{p0}\right)}{log\varepsilon_{\omega }}$$

Other important parameters can be obtained by EIS. For instance, the coating damage index and coating delamination extent can be calculated from a simple analysis of impedance values at low frequency, the phase angle at high frequency, and the areas under the Bode modulus plots. Additionally, the breakpoint frequency (*f_b_*), which is the frequency at −45° phase angle, is a useful parameter to investigate the performance of a polymeric barrier over time. The EIS data of a multilayer automotive system (phosphate, electrodeposition, polyester/melamine, and acrylic topcoat) on steel were used to calculate the coating detachment index by three different methods after the stone chipping test [70]. The results showed good agreement between the images of the aged surfaces and the EIS analysis. To estimate the coating delamination, the calculation of the capacitive and resistive areas under the Bode diagrams as well as the breakpoint frequency were the most suitable parameters since they consider a broad frequency domain. To this end, the authors divided the area under the Bode plot into two regions of *A*_1_ and *A*_2_, representing the capacitive and resistive components, respectively [70]. The damage function (DF) was obtained using Equation (4):

(4) $$DF\left(\%\right)=\left(1-\frac{A_{2}+A_{1}}{A_{1}}\right)\times 100$$

Similarly, the coating delamination index (CDI) can be calculated at a single frequency (ǀ*Z*ǀ_0.1 Hz_) through Equation (5), where *Z_i_* is the impedance value at the initial stage and *Z_f_* corresponds to the impedance at the final stage of aging. This approach was used to evaluate the interfacial bond destruction of the epoxy coatings modified with amino-functionalized graphene oxide that were deposited on mild steel substrates [77]. The calculations allowed to identify an improvement in the adhesion of the modified coatings in relation to the reference material. Furthermore, lower electrolyte diffusion was observed due to the presence of graphene oxide nanosheets dispersed in the epoxy matrix [77].

(5) $$CDI\left(\%\right)={\left(\frac{Z_{i}-Z_{f}}{Z_{i}}\right)}_{0.1\mathrm{Hz}}\times 100$$

Typically, EIS measurements are obtained in an electrochemical cell using an experimental setup of three electrodes: a working electrode (coated substrate), a reference electrode (e.g., SCE, Ag/AgCl), and a counter electrode (e.g., Pt wire or mesh, carbon). These electrodes are placed in a horizontal or vertical electrochemical cell in the presence of a conductive electrolyte and connected to a potentiostat/galvanostat that is attached to a computer that records the data. In the center of Figure 8, the basic configuration of a vertical electrochemical cell and its electrodes is represented [71].

### 2.3. Advanced Electrochemical Analyses

The previous section was devoted to electrochemical measurements that represent an average of the electrochemical processes taking place on the entire surface of the working electrode. Below, this review describes the main techniques developed to detect the local corrosion reactions of active systems, which are documented with successful examples of their application for anti-corrosion polymeric coatings.

#### 2.3.1. Localized Electrochemical Impedance Spectroscopy (LEIS)

The LEIS technique is based on the same principle as EIS. It measures the ratio between the applied AC voltage and the measured AC density between a sample and a counter electrode. Essentially, LEIS relies on the measurement of the local output current, which allows the calculation of the local impedance [18,78]. As a spatially resolved technique, the main applications of LEIS for polymeric coatings include tracking corrosion inhibition as well as the initiation and propagation of the delamination. While EIS provides only global surface-averaged responses, LEIS can provide highly accurate information on isolated defects (pits or scratches), which, in turn, corresponds to the global impedance due to the possibility of simultaneous measurements. LEIS is also a powerful technique to study substrate reactions underneath the coating. However, the size of the probe and the distance between the probe and substrate are important parameters that can affect the resolution of the results. It has been reported, for instance, that smaller probes provide better spatial resolutions [78].

The measurement of local impedance in the analysis of well-defined areas of polymeric coatings allows the obtaining of 2D or 3D color maps of defects and the observation of their regeneration when the film contains corrosion inhibitors/self-healing substances. Normally, LEIS maps are displayed in the admittance formalism, which is the inverse of the impedance (*Y = Z*^−1^). In other words, for an active inhibition system, it is expected that a decrease in Y values will be observed over time.

**Figure 9:**
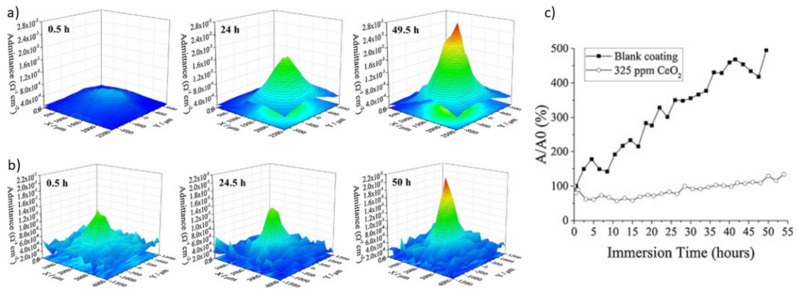
LEIS admittance mapping over an artificial defect on the surface of (**a**) blank and (**b**) CeO_2_-modified coating after 0.5 h, 24.5 h, and 50 h immersion in 0.005 M NaCl; (**c**) Ratio between the measured admittances during LEIS and the first registered admittance for each sample. Reproduced with permission of Elsevier [79].

**Figure 10:**
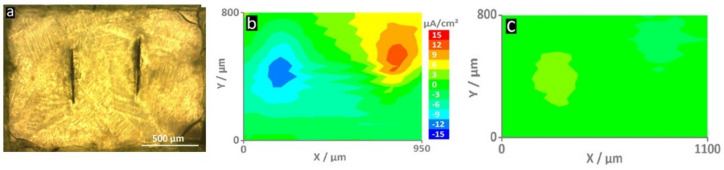
These optical micrographs (**a**) show the self-healing surface with two vertical screwdriver-blade indentations that form the defects through the coating. The map of the reference sample (**b**) shows high current densities (±12 μA/cm^2^)—an indication of an actively corroding sample. On the contrary, the sample with MLDH (**c**) shows much smaller current densities and thus corrosion inhibition [82].

Calado et al. investigated epoxy coatings modified with aminopropyltriethoxysilane (APTES) to improve the adhesion between the magnesium substrate AZ31 and CeO_2_, which is used to provide corrosion inhibition [79]. The LEIS tests were performed by submerging coatings with an artificial defect of about 750 µm in size in a 0.005 M NaCl solution, thus measuring blank and modified material after 0.5 h, 24 h, and 50 h of immersion. According to the maps presented in Figure 9a,b, the evolution of the admittance values showed a different behavior for each coating. For the blank material, the admittance showed an increase of one order of magnitude after 50 h of immersion compared to the initial stages (Figure 9a). However, the modified CeO_2_-coating on the magnesium substrates delayed the onset of corrosion by up to 35 h and showed much lower admittance variation (Figure 9b) [79]. The CeO_2_ inhibitory activity in damaged coatings was thus confirmed by LEIS, as shown in Figure 10c, with the admittance evolution over time compared to its initial value, reflected as a stable behavior in the presence of 325 ppm CeO_2_ [79].

The self-healing action of cerium salts loaded within CaCO_3_ microspheres was also confirmed in epoxy coatings on AA2024 substrates using LEIS [75]. With the addition of CaCO_3_ as pH-sensitive containers, a decrease in admittance values over time (46 h) was observed in artificially damaged coatings immersed in a 1 mM NaCl solution. Combining LEIS (spatially resolved) and EIS results, the authors demonstrated the efficiency of inhibition provided by cerium salts in large-scale (6 mm × 0.3 mm) defects [75]. The action of CeMo nanocontainers loaded on epoxy–silicate films was confirmed by LEIS elsewhere [80]. This study used a 5 mM NaCl solution as an electrolyte during the measurement of artificially scratched films. The temporal evolution of admittance values showed that the reference coating exhibited an increase in corrosive activity, while the coating modified with the inhibitor showed a decrease in corrosion. The behavior observed by LEIS agrees with the EIS data, pointing to the reproducibility of the self-healing activity [80].

#### 2.3.2. Scanning Vibrating Electrode Technique (SVET)

The use of a micro-tip (less than 20 µm) to detect the current distribution in solutions became an important tool for studying polymeric coatings and is based on the study carried out by Lilard et al. in 1992 [18]. The main outputs of SVET are ionic current density maps measured in the micrometer range of a corroding system that allow the visualization of the oxidation reactions at the anode sites and the reduction reactions at the cathode sites [71]. The measurements are performed in an electrochemical cell composed of a piezo-oscillator system that produces vibrations from the microelectrode that sweeps the coating surface (typically with artificial defects), a platinized reference electrode in the shape of a sphere (Pt/Ir: 80%/20%), and a signal amplifier [71]. After immersion in a conducting electrolyte, the current density distribution maps are generated as a result of DC potential gradients in the solution. SVET has been widely used to study active systems with corrosion inhibitors or self-healing agents; however, its application is limited to electrochemically active systems where DC currents flow in the solution so that corrosion reactions underneath intact coatings cannot be assessed [18]. Moreover, probe platinization and calibration can be laborious, not to mention its fragility and short lifespan. Nonetheless, SVET maps can be obtained in parallel with pH maps by coupling the scanning ion-selective electrode technique (SIET), thus making it a powerful tool to simultaneously analyze local current and pH changes [81].

With increasing interest in active and smart anti-corrosion systems, the number of publications reporting the use of SVET has grown significantly in recent years. For example, Lutz et al. presented SVET maps of a shape-recovery coating based on acrylated polycaprolactone polyurethane applied on hot-dip galvanized steel containing the corrosion inhibitor 2-Mercaptobenzothiazole (MBT) loaded in layered double hydroxide (MLDH) [82]. The system containing the MLDH was compared with the reference material. As shown in Figure 10, the release of MBT at the active corrosion sites resulted in a significant decrease in current densities (from ±12 µA/cm^2^ to ±2–3 µA/cm^2^). The ionic current density maps were recorded after 4 h of immersion at 100 μm from the surface [82].

The results provided by SVET improve the understanding of the inhibition mechanisms of corrosion inhibitors. The activity of inorganic compounds such as cerium salts in epoxy–silane hybrids on magnesium alloy AZ31 [83] or organic agents such as 8-hydroxyquinoline (8HQ) in sol-gel films prepared from zirconium (IV) propoxide on AA2024 [84] was unveiled with the use of SVET. In the case of cerium salts, the Ce^3+^ cations react with the OH^−^ anions released in the cathodic zone to form Ce(OH)_3_, a very stable compound at 25 °C (K_sp_ Ce(OH)_3_ = 6.3 × 10^−24^), which inhibits cathodic activity and, consequently, the corrosion processes [61]. On the other hand, the action of 8HQ is based on the formation of chelate complexes with copper or aluminum, suppressing the initial stages of localized corrosion attacks [84].

#### 2.3.3. Scanning Ion-Selective Electrode Technique (SIET)

The setup of SIET measurements is similar to that of SVET measurements. However, in this technique, an ion-selective microelectrode, in the form of a glass capillary, is used to scan the surface in a 3D computerized system. The microelectrode is filled with a liquid membrane capable of measuring ions down to the picomolar level, the electrolyte, and an Ag/AgCl wire as a reference electrode [85]. This membrane is composed of different ionic species that can be chosen, for example, according to the pH range to be studied. SIET is a powerful technique for the evaluation of corrosion inhibitors’ efficiency by identifying the pH gradients in active systems with good lateral resolution. Although the possibility of filling glass capillaries with different membranes is an advantage, working with microelectrodes is not an easy task because they are brittle and their lifetime is limited to a few hours or a maximum of one day [85]. In addition, silanization of the capillary is required to hold the membrane. When this step is not properly performed, the membrane leaks easily, making the operation time-consuming and difficult.

**Figure 11:**
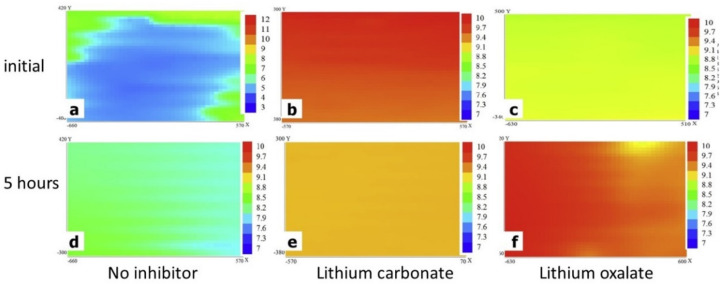
SIET pH scans of scribed area (1 mm × 2 mm): (1) for the initial stage after 5 min exposure for model coatings with (**a**) no inhibitor, (**b**) lithium carbonate, and (**c**) lithium oxalate and (2) after 5 h exposure with (**d**) no inhibitor, (**e**) lithium carbonate, and (**f**) lithium oxalate. Reproduced with permission of Elsevier [87].

Knowledge of the local surface pH can help identify the mechanisms of corrosion inhibitors in specific regions, considering the local pH variations that occur in the anodic and cathodic regions (see Figure 2). It is also of great importance to choose corrosion inhibitors appropriately according to the working pH range and substrate [86]. Visser et al. observed significant pH variations in scribed areas of polyurethane coatings on AA2024 by incorporating different lithium salts (carbonate and oxalate) [87]. Some differences are seen in Figure 11, where SIET maps depict the hydrogen concentration in the scribes (1 mm wide) using a membrane that is active in the pH range of 5.5–12.0 in a 0.05 M NaCl solution. An increase in local pH (from 5 to 8.8) was observed for the reference sample, which can be attributed to the natural pH variation in the studied aluminum alloy [87]. However, a sharp difference in alkalinity was observed after 5 min and 5 h for both inhibitors. This clear contrast was accompanied by the formation of a protective layer observed by SEM and XPS and allowed the mechanism of action to be determined for each salt and its conjugated bases, namely the faster leaching of lithium carbonate and the formation of a pseudo-boehmite layer from aluminum hydroxide under alkaline conditions [87].

Other studies with cerium-based corrosion inhibitors reported interesting results for the SIET measurements. For example, the addition of CeO_2_ [79] or cerium phosphate [83] to epoxy–silane coatings on an AZ31 magnesium alloy provided indications of a local pH shift induced by the reaction between Ce and hydroxyl ions in the cathodic regions [79,83]. In addition to investigations of the self-healing effect, SIET maps are useful for comparative studies of metallic alloys, such as the analysis of corrosion mechanisms for different magnesium alloys, such as AZ31 and ZE41 [88]. The higher corrosive activity of AZ31 observed by SIET and SVET was reflected in the more pronounced differences in pH and current density values (80 µA cm^−2^ for ZE41 vs. 120 µA cm^−2^ for AZ31) [88].

#### 2.3.4. Scanning Kelvin Probe (SKP)

A variant of atomic force microscopy (AFM), scanning Kelvin probe (SKP), can be obtained by applying the non-contact scan mode to generate surface topography maps and potential distribution of the work function (or Volta potential). A decrease in the Volta potential can be considered as an increased tendency for electrochemical reactions [89]. SKP is widely used in coatings and corrosion studies, but it can also be applied to measurements of the composition and electronic state of a solid’s local structure. These other solids include catalysts, semiconductor doping, dielectric materials, the investigation of intermetallic in alloys, and others [89]. Its major advantages include measuring the electrode potential without touching the surface, since the reference electrode is connected to the sample through a metallic wire and separated by a dielectric medium (for instance, under a vacuum). SKP, also known as Kelvin probe force microscopy (KPFM), is a versatile tool for the analysis of polymeric coatings and can be operated in open-air, humid air, or by using a single drop of electrolyte. Although it provides limited spatial resolution (about 100 µm for a 150 µm probe diameter), resolutions in the nanometer range can be achieved when it is coupled with an AFM [81].

SKP is widely applied in the study of conducting polymeric coatings (CPCs). The combination of electronic properties of semiconductors and the processing of conventional polymers makes this type of material an interesting alternative for anti-corrosion applications [49]. Various mechanisms have been proposed to explain their anti-corrosion properties, such as controlled inhibitor release, barrier properties, and anodic protection [50]. In particular, the latter mechanism is known due to observations of the oxygen-reduction reactions shifting from the coating/substrate interface to the coating/electrolyte interface, thus preventing the delamination of the coating [50]. On that basis, SKP is a useful tool to investigate the delamination kinetics of CPCs. By using SKP, for example, it was shown that small cations from the electrolyte can permeate polypyrrole-based films, causing faster delamination of the coating [50]. 

Bai et al. observed a significant reduction in the delamination rate by adding carbon black (CB) to poly (3,4-ethylene dioxythiophene) nanoparticles (PEDOT) and polyvinyl butyral (PVB) coatings on zinc substrates [90]. The PEDOT/CB/PVB composites slowed the cathodic delamination to 10 µm h^−1^ and even stopped after 3 days of immersion in 1 M KCl [90]. The work of Yin and co-authors on polypyrrole coatings modified with organic corrosion inhibitors such as β-Cyclodextrine (β-CD), benzotriazole (BTA), or 8-Hydroxyquinoline (8-HQ) used SKP to evaluate their self-healing performance [91]. Excellent passivation was observed by monitoring the corrosion potential and SKP profiles for the artificially damaged polypyrrole–BTA coating. This performance is proposed to be a consequence of the anodic polarization by the re-oxidizing polypyrrole and the BTA activity that fully restored the delaminated interface [91]. In a similar study, the authors compared the mobility of different corrosion inhibitors through the partially reduced polypyrrole coating, and they found higher activity for cationic inhibitors due to the cation-permselectivity of this polymeric matrix [92]. Electrochemical passivation through re-oxidation of polypyrrole was also observed, resulting in potential shifts of up to 700 mV, an effect potentiated by the combination of conductive polymers and corrosion inhibitors [92]. 

**Figure 12:**
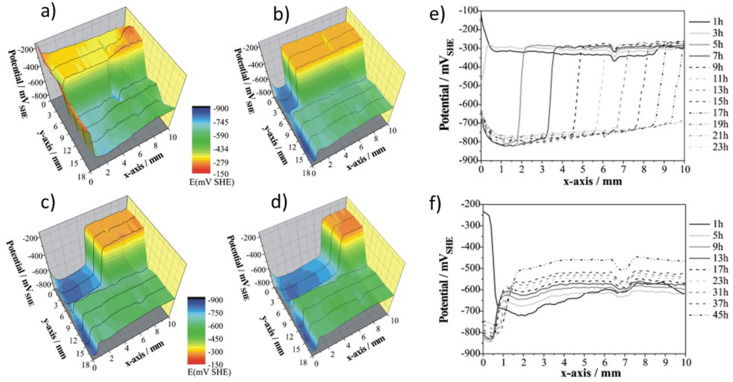
(**a**–**d**) Potential profiles during delamination for a non-pigmented, transparent polymeric film on electrogalvanized (EG) steel with (y-axis: from 6 to 18 mm) and without (y-axis: from 0 to 6 mm) a conversion layer at the interface during the cathodic delamination process as measured by the HR–SKP; (**a**) 1 h, (**b**) 5 h, (**c**) 9 h and (**d**) 13 h. SKP line scans of polymer-coated EG steel during the cathodic delamination process: (**e**) alkali-cleaned zinc surface without conversion layer and (**f**) conversion layer–coated zinc surface. Reproduced with permission of Elsevier [93].

**Figure 13:**
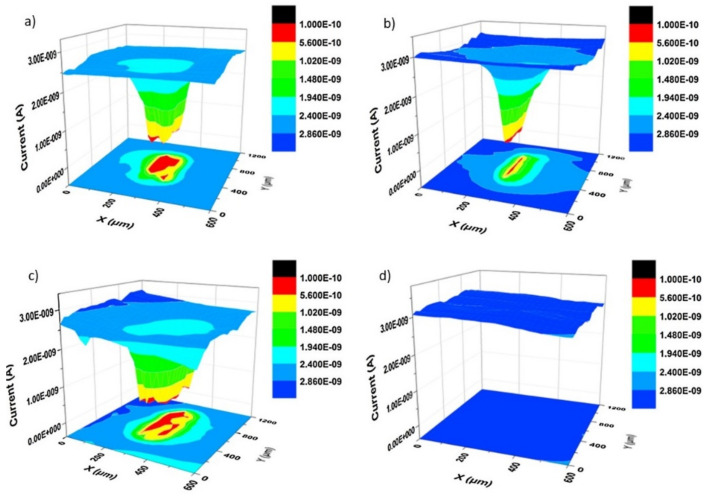
SECM measurements of the coating defect area before and after the self-healing process: (**a**) original defect in wax-free coating, (**b**) defect in the wax-free coating after two-step healing, (**c**) original defect in SMC coating, and (**d**) defect in SMC coating after two-step healing. Reproduced with permission of Elsevier [41]. Some like 1.000E-10 mean 1.000 × 10^−10^.

A successful example of SKP being applied for the study of anti-corrosion coatings is an electrochemical study of the cathodic delamination of galvanized steel coated with epoxy–amine films (~10 µm) [93]. Figure 12 shows the 3D cathodic delamination process of the coating after 1 h to 13 h (Figure 13a–d). It is possible to observe the drop in potentials from 500 mV to 150 mV from the intact coating towards the uncoated zinc, respectively. From a two-dimensional view, Figure 13e and Figure 13f compare the delamination of coatings on steel substrates with and without a zinc conversion layer, respectively. The line scans prove the superior corrosion protection provided by the conversion layer, retarding the delamination rate (Figure 13f) [93].

SKP is an important tool for adhesion studies as the delamination rate can be assessed at the metal/coating interface. Posner et al. showed that a small bubble-shaped defect under the epoxy–amine coating on a Zn-electrogalvanized steel substrate served as an electrolyte reservoir that initiates the cathodic delamination process [94]. The SKP results demonstrate that the mechanical and visual degradation processes are preceded by non-visual cathodic delamination caused by electrochemical reactions. The average rate of delamination with an electrolyte pressure of 10 kPa was 42 ± 1 µm/h. In the studied area, the film was completely removed within 69 h [94]. In another work, styrene/acrylate films with various glass transition temperatures (T_g_) ranging from −40 °C to 40 °C were prepared on an iron substrate. The delamination was fastest for the films with the highest T_g_ [95]. Although higher T_g_ values resulted in increased polymer crosslinking, the authors attribute this outcome to the structure having a higher number of pores and defects, thus facilitating the transport of the electrolyte [95].

SKP can also be used for the analysis of the filiform corrosion mechanisms. The delamination mechanism varies under different conditions such as the presence of an electrolyte or atmosphere at 95% humidity. The defects can act as an anode when immersed in the NaCl electrolyte. However, in the presence of moisture, they work as a cathode, resulting in the formation of anodic zones around them [96]. Both processes are spread over the surface, resulting in film delamination and widespread corrosion [96].

#### 2.3.5. Scanning Electrochemical Microscopy (SECM)

SECM combines high electrochemical sensitivity with spatial resolution, resulting in a powerful tool for characterizing a variety of electrochemical reactions in active coating systems. The principle of operation of SECM is simple: a microelectrode (Pt wire) scans the surface of a sample immersed in electrolyte, providing information on the topography and/or redox reactions taking place at the interface tip/sample [71]. One of the main advantages of SECM is its simultaneous characterization of the topography and redox activity of immersed samples. In other words, it is possible to obtain information about the reactions that take place on the surface and in the space between the tip and the sample [71]. Its usefulness can be attributed to its high lateral resolution when compared to SVET due to the wide range of electrode sizes, allied with the possibility of combining it with other techniques in several modes of operation. As the main drawbacks of SECM, one can mention the need for a bipotentiostat to polarize the microelectrode and sample, the dependence of the resolution on the microelectrode size and distance from the surface, and competing redox reactions that can occur at the probe and surface at the same time [71,81].

SECM was used to study the addition of decavanadate-intercalated layered double hydroxide (LDH-VS) loaded into sol-gel films on AA2024 aluminum alloy. The results of artificially scratched samples show the absence of lateral delamination towards the film side, probably caused by the structural and adhesion improvement provided by LDH-VS [97]. In another report, the recovery of the barrier properties of shape-memory epoxy coatings (50 ± 5 µm thickness) modified with carnauba wax microparticles was also observed by SECM [41]. Figure 13 shows a 3D comparison between the current variation of the reference coating (Figure 13a,b) and the modified film (Figure 13c,d) after two healing steps (heating to 65 °C and 90 °C). The red zone corresponds to the most severely corroded area in the coating defect. However, the ~40 µm cut was completely repaired when in the presence of wax since the current values were substantially decreased (Figure 13d) [41]. This result highlights the effectiveness of SECM for studies of high-performance self-healing coatings. The information provided, advantages, and limitations of the techniques covered in this section are summarized in Table 1.

### 2.4. Theoretical Approaches

**Figure 14:**
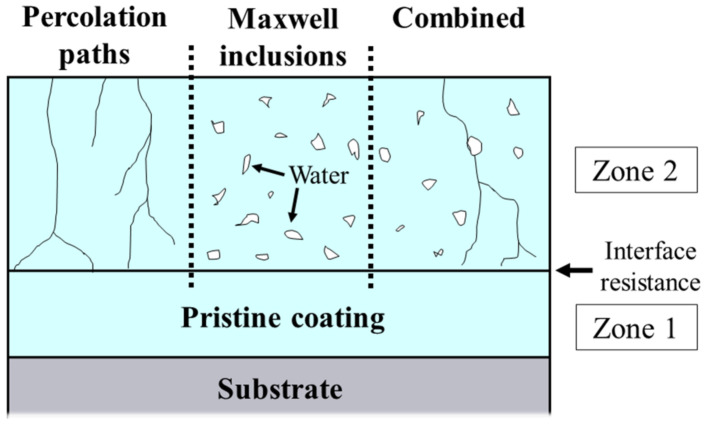
The electrical resistivity and relative dielectric constants for the diffusion-affected zone are modeled in three ways: first, as percolation paths which act as channels to deliver water to the interface and advance the diffusion-affected zone; the second model assumes a Maxwell-type two-phase system where water fills voids within the coating and coating inclusions; and the third model combines inclusions, holding most of the water, with a few channels that connect the water inclusions. Adapted from reference [101].

Theoretical approaches represent an active and important research area in the study of anti-corrosion polymeric coatings. Currently, some investigations based on modeling are focused on developing tools capable of predicting corrosion in practical systems. However, this does not replace the fundamental studies, theoretical calculations, and computational modeling that have been carried out by research groups worldwide. Many works have demonstrated the efficiency of combining experimental measurements with electrochemical data modeling to access crucial information about a studied system. It is worth mentioning some of the main applications of theoretical approaches, which include:

- Estimating water diffusion (or water uptake) [76,101,102];
- Determining the resistivity/conductivity profiles [74,102,103];
- Analyzing the non-ideal capacitive behavior of coatings [73,74];
- Monitoring and quantifying physical and dielectric properties [73,74,102];
- Comparing water and ion diffusion rates [74];
- Investigating polymer swelling [104];

The permeation of an electrolyte into a polymeric film has been the subject of many studies and some models have been proposed such as (i) percolation paths, (ii) Maxwell inclusions or the presence of voids, and (iii) the combination of both [101]. Although there is no consensus on the electrolyte uptake process, several researchers agree on the existence of a non-homogeneous uptake composed of an outer permeation layer at the coating/solution interface and an immaculate inner layer at the coating/substrate interface [28,74,101,105]. According to this model, the dielectric constant in the permeated region increases considerably, going from the dielectric constant of the dry coating to that of water (*ε_ω_* = 78.3). Using different models, Figure 14 shows the formation of the two permeation zones with a 2D view of a coating cross-section.

The assumption of a two-layer permeation front comes from calculations of resistivity (*ρ*) and dielectric constant (*ε*) of coatings, along with their thicknesses. The main advantage of calculating these values compared to monitoring the modulus of impedance (ǀ*Z*ǀ) is the ability to obtain the effective diffusion rate and water saturation profile before the film breaks. This information is not readily available from the evolution of the ǀ*Z*ǀ data. Therefore, the decay of *ρ* and *ε* can be used to predict the effective lifetime of a coating long before the appearance of corrosion pits [101]. In addition, modeling helps to identify and assign distinct time constants, mainly calculated from the phase diagram over time. In a recent study, a theoretical approach was used to understand the influence of water on the aging of a polyester/melamine/epoxy system’s physical structure [104]. It was found that the time constant (*τ*) obtained from the impedance diagrams decreased due to the plasticizing effect of water on the polymer chains [104].

Although EIS data–fitting using Randles circuits in series is widely used, it is difficult to establish a relationship between the physical properties of the film during permeation and the constant phase element (CPE) normally employed in this type of modeling [28,75]. Hence, another advantage of theoretical approaches is the ability to approximate the real complexity of systems when they are immersed in an electrolyte. This is particularly useful for analyzing high-performance coatings because, in these cases, the changes in the EIS diagrams are almost imperceptible for a long time [27,28]. From this perspective, investigations have been conducted to identify the distribution of resistivity and dielectric constant by the regression of EIS data using mainly the power law and the model proposed by Young [28,73,74,103,106].

**Figure 15:**
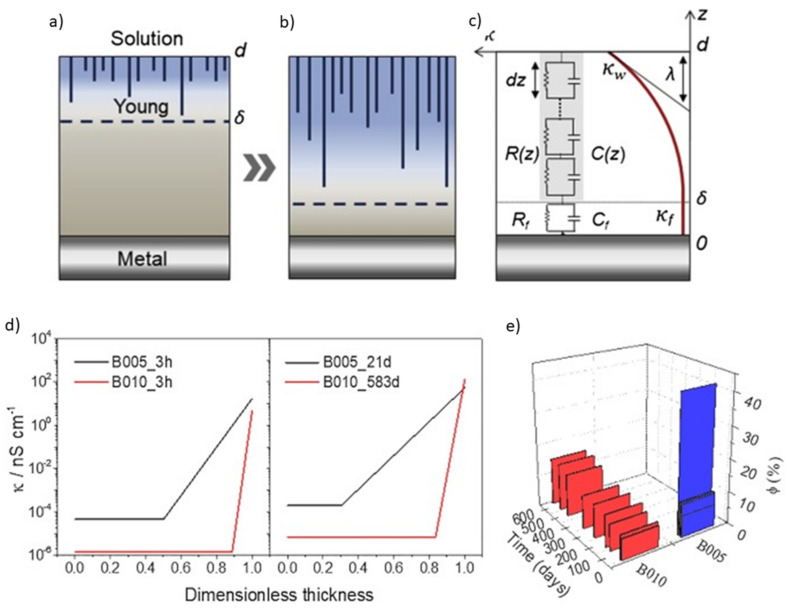
Representation of electrolyte permeation through a coating according to the two-layer model: (**a**) the initial stage of contact with the solution; (**b**) after long-term exposure; (**c**) the corresponding continuous time-constant distribution, resulting from an exponential conductivity profile along the z-axis in the water uptake zone; (**d**) profiles of conductivity vs. dimensionless thickness for different immersion times in 3.5% NaCl for B005 (3 h and 21 days) and B0.1 (3 h and 583 days) coatings; (**e**) time dependence of water uptake. Reproduced with permission of Elsevier [28].

Despite the good agreement between the experimental data and resistivity profiles of dry coatings calculated by the power law, this model is not suitable for long immersion times due to the straight constant phase angle profiles [74]. Young’s approach, in turn, considers an exponential decay of resistivity along with the thickness, forming the so-called “two-layer model” [74,106]. This model relies on the diffusion of water in channels or voids in the film, creating a permeation front that progressively eliminates the inner dry layer when the electrolyte reaches the substrate [28,73,74]. In this case, the total system impedance is given by an equation that sums the dry and wet layer impedances, as represented in Equation (6) [28,73,74]:

(6) $$Z=R_{S}+\frac{R_{f}}{1+j\omega R_{f}C_{f}}-\frac{\lambda }{j\omega \varepsilon_{0}\varepsilon_{w}}ln\left(\frac{\kappa_{f}+j\omega \varepsilon_{0}\varepsilon_{w}exp\left(\frac{\delta -d}{\lambda }\right)}{\kappa_{f}+j\omega \varepsilon_{0}\varepsilon_{w}}\right)\;$$

where *R_S_*, *R_f_* and *C_f_* represent the solution resistance, film resistance, and film capacitance, respectively. λ refers to the rate of conductivity decay. *κ_f_*, *ε*_0_, and *ε_w_* correspond to the conductivity at the metal/coating interface, the vacuum permittivity, and the permittivity in the uptake zone, respectively. Finally, *d* and *δ* represent the thicknesses of the coating and the inner layer, respectively. Using the parameters obtained by this model, it is possible to plot the conductivity or resistivity profiles of high-performance coatings and compare them with the water uptake values obtained by the Brasher–Kingsbury (BK) relationship [74,107], as shown in Figure 15.

In Figure 15a–c, the two-layer model described by Young is graphically represented, along with a picture of the continuous distribution of time constants (R_C_) throughout the thickness and the parameters used in Equation (6) [28]. Using this model, the authors were able to plot the dimensionless conductivity profile and they observed a very small variation during 583 days for the B010 film due to a slow permeation of the inner layer. This indicates that the acrylic coating applied to a steel substrate (~10 µm) has a highly crosslinked structure that delays the permeation and swelling of the polymer chains [28]. At the same time, using the BK model to compare the preserved layer’s thickness values showed good agreement between the values of sample B010, indicating that Young’s model is suitable for analyzing the permeation of high-performance polymeric coatings in harsh conditions [28].

## 3. Concluding Remarks

An overview of the main electrochemical techniques employed for protective coating characterization reveals that in-depth structural knowledge combined with electrochemical analysis are the key factors in developing high-performance coatings. An interdisciplinary approach is necessary to obtain an efficient barrier with chemical, mechanical and thermal stability, corrosion inhibition, self-healing ability, and other desired properties. However, predicting corrosion remains a challenge, both for theoretical and experimental studies. Current efforts are directed towards developing practical and efficient tools based on machine learning (ML) and data science applied to corrosion studies to estimate the lifetime of polymeric films in-situ. Although significant advances have been made in the field, it is still necessary to consolidate devices that can be applied to most systems that are susceptible to pitting corrosion, hydrogen embrittlement, crevice corrosion, galvanic, fatigue, and filiform corrosion, among others.

Looking to the future, the main strategies for developing advanced coatings, theoretical simulations, and cutting-edge technologies point to multifunctional green materials of low complexity and high efficiency. The development of eco-friendly coatings is in full agreement with circular economy guidelines, where reuse is an important pillar of sustainable consumption. Efforts have been redirected to the replacement/reduction of organic solvents and the development of water-based technologies, powder coatings, UV-curable coatings, non-toxic corrosion inhibitors, and bio-based polymers.

The combined analysis of electrochemical data through machine learning, in situ spectroscopic techniques, and the imaging of the aged surface is a promising approach for studying coating degradation. Devices that combine theoretical and experimental knowledge relative to composition, structure, and electrochemical properties are persued to evaluate a coating’s performance and predict failures. The information collected from this apparatus will be beneficial for developing new coatings that provide extended safety through the use of eco-friendly materials. To this end, advances in techniques for the characterization of anti-corrosion polymeric coatings point towards a comprehensive and combined investigation. In other words, an interdisciplinary approach between physical, chemical, and theoretical methods is needed to investigate complete systems and monitor the evolution of flaws. Finally, the ability to understand the structure at the atomic level controlling it on a molecular scale, and the complex phenomena related to coating failure merge different fields of fundamental and applied research, thus representing a valuable exchange of knowledge between the academy and industry, including, but not limited to, the automotive, aviation, construction, and maritime industries.

## Figures and Tables

**Table 1:** Information provided, advantages, and limitations of electrochemical techniques used for the characterization of polymeric coatings.

Technique	Information Provided	Advantages	Limitations	Ref.
*Open circuit potential*	Zero-current potential, equilibrium potential, or rest potential of a working electrode Indication of the corrosion protection	Non-destructive Easy to operate Cost-effective and fast measurement Quick estimation of the corrosion protection The results can be compared with EIS measurements Tracking OCP can reflect the changes in polymer permeation and corrosion of the metal	The values can be affected by several factors, such as the resistance of the film, pH, presence of corrosion products, alloy composition, an oxide layer, etc Electrolyte diffusion and corroding systems result in large fluctuation of values Changes in the ratio of cathodic and anodic areas lead to fluctuations	[5,6,7,46]
*EIS*	Imaginary and real impedance over a frequency range Barrier properties of the coating	Non-destructive Monitors the degradation of immersed coatings over time Monitors the passivation by self-healing or inhibition of smart coatings Easy to compare different samples under the same conditions Quantitative information about charge transfer resistance and double-layer capacitance Provides the input values to calculate coating capacitance, resistance, and water uptake Good agreement with DC techniques	Fitting models can be complex Difficulties in the interpretation of time constants The presence of capacitive or inductive loops can lead to misleading interpretations Difficulties in the interpretation of diffusing or corroding systems Prior knowledge about the coating and corrosion system is required Local impedance measurements are not provided, the results correspond to an average of the surface	[20,98,99]
*LEIS*	Local ac impedance and admittance Local current, capacitance, and resistance Solution current density	Possibility of simultaneous measurement of local and global impedance Study of delamination of coatings Evaluation of corrosion inhibition and self-healing performance Assessing the corrosion underneath insulation coatings Measurement sensitivity on the order of 1 nV Measurements can be carried out at a fixed frequency or over a range 2D–3D color gradient maps	The resolution depends on the size of the electrodes/sensor and the distance between the electrode and sample Stray inductance High-frequency inductive behavior when small probes (tens of µm or less) are employed	[27,71,81]
*SVET*	Potential difference and current density between probe and substrate Local maps of current densities in the micrometric range Location of anodic and cathodic zones	In-situ measurements of localized corrosion Non-destructive method Evaluation of corrosion inhibition and self-healing performance Monitoring of inhibition activity in a coating defect Information about swelling and formation of defects Possibility of simultaneously using SVET and SIET in a single instrument Real-time mechanistic information 2D–3D color gradient maps The vibration of the probe helps minimize concentrations gradients	Tip microelectrode is costly and fragile Microelectrode requires platinization Artifacts between the probe and sample, the capacitance of the electrode tip, probe vibration speed, improper platinization of the tip Need for probe calibration Suitable for active corroding systems only High conductivity electrolytes cannot be used Evaporation of the electrolyte to the air can cause artifacts The high amplitude of vibration Diluted electrolytes are preferred to maximize the potential difference	[27,71,78,81]
*SIET*	Real-time measurements of local pH Ion concentration	Evaluation of corrosion inhibition and self-healing performance in coating defects Possibility to simultaneously use SIET and SVET in a single instrument Easy to measure ion concentration Glass capillary allows different types of membranes to analyze different ions and pH ranges Assessment of corrosion mechanisms Good lateral resolution (tip size ranges from 0.1 to 5 µm)	Time-consuming and difficult operation Glass capillary is brittle and its preparation is labor intensive Silanization of the capillary is required to hold the membrane The membrane leaks easily The short lifespan of the capillary (max. 1 day) Fouling and damage to the ion-selective electrode Resolution can be compromised by the distance between the electrode and sample, membrane column length Difficult to reproduce the results	[81,85]
*SKP*	Work function (Volta potential) Capacitive height measurements Delamination profiles	Evaluation of corrosion inhibition and self-healing performance Studies of the delamination of coatings Can be operated in a vacuum, open-air, humid air, or using a drop of electrolyte Pitted and roughened surfaces can be measured (when coupled with AFM) Fast experiments	The probe needs calibration Low resolution Needs to be combined with AFM to achieve higher spatial resolution (SKPFM approx. res. is 0.1 µm) Work function decreases on very rough surfaces	[27,81,89,100]
*SECM*	Current maps Electrochemical activity and/or topography of the substrate (moving the probe in the x or y direction) Electron transfer kinetics (moving the probe in the z-direction)	Evaluation of corrosion inhibition and self-healing performance Several modes of operation Can be combined with other techniques Multiple operational modes Bipotentiostat provides independent control and sensing for the substrate and ultramicroeletrode	Sometimes needs a redox-active mediator A bipotentiostat is required Proper design of the measurement is not trivial Possible contamination of the microelectrode An adequate scan rate must be selected to assure the steady-state	[71,81]

## Data Availability

Not applicable.
